# Nasal Reshaping Using Barbed Threads Combined With Hyaluronic Acid Filler and Botulinum Toxin A

**DOI:** 10.1111/jocd.70047

**Published:** 2025-02-12

**Authors:** G. Ziade, R. Saade, D. Daou, D. Karam, A. Bendito, M. Tsintsadze

**Affiliations:** ^1^ Department of Otolaryngology—Head and Neck Surgery Lebanese American University School of Medicine Beirut Lebanon; ^2^ Department of Anesthesia American University of Beirut Medical Center Beirut Lebanon; ^3^ Faculty of Medicine Holy Spirit University of Kaslik Jounieh Lebanon; ^4^ Martina Children's Hospital Stockholm Sweden; ^5^ Faculty of Medicine BAU International University Batumi Batumi Georgia

**Keywords:** Aptos threads, botulinum toxin, hyaluronic acid, noninvasive, poly‐l‐lactic acid, rhinoplasty

## Abstract

**Background:**

Rhinoplasty is a prominent procedure in facial aesthetics, but extended surgical downtime remains its significant limitation. Nonsurgical rhinoplasty has gained popularity as an alternative, offering significant aesthetic improvements with minimal downtime. This study evaluates the clinical efficacy and safety of three nonsurgical rhinoplasty techniques combined, including hyaluronic acid (HA) filler, botulinum toxin type A (BTX), and barbed lifting threads.

**Methods:**

A total of 85 patients were included into three groups: 63 subjects received HA filler and BTX injections, 9 subjects received barbed threads followed by BTX injections, and 13 received threads followed by HA filler and BTX injections. Patients assessed their satisfaction 1 month and a year posttreatment as well as adverse effects 48 h and 1 week after treatment using FACE‐Q questionnaire for nose.

**Results:**

Both 1 month and a year after treatment, the triple combination of barbed threads with HA filler and BTX demonstrated superior nose FACE‐Q scores compared to the two other groups, showing highest patient satisfaction and minimal efficacy decay over time. No significant difference in FACE‐Q scores was observed between the two groups receiving HA filler + BTX and barbed threads + BTX at either 1 month or a year posttreatment. Adverse effects were reported by subjects in all three groups 48 h posttreatment and completely resolved by 1 week after treatment.

**Conclusion:**

Combining nonsurgical rhinoplasty techniques—such as lifting threads, HA fillers, and botulinum toxin—can deliver effective aesthetic improvements with minimal downtime. Among these, the triple combination of HA filler, BTX, and lifting threads results in the highest and most sustained patient satisfaction, lasting no less than 1 year.

## 1 Introduction

The nose, as a central feature of the face, plays a pivotal role in defining facial aesthetics. Key anatomical elements such as the nasal dorsum, tip, alar base, and internal nasal valve collectively contribute to its overall appearance [1]. Consequently, rhinoplasty is among the most performed procedures worldwide and remains a significant topic for scientific discussions, hands‐on courses, and innovative surgical laboratories [2].

Despite significant advancements in nasal surgical techniques, the extended surgical downtime remains a major limitation for patients. As a result, nonsurgical rhinoplasty has gained considerable appeal among both aesthetic physicians and patients over the past two decades. Nonsurgical rhinoplasty is being evaluated based on procedure's safety, significance of aesthetic improvement, as well as its convenience as an in‐office treatment [3, 4].

The development of new hyaluronic acid dermal fillers with favorable efficacy and safety profiles has significantly increased the popularity of nonsurgical rhinoplasty (NSR) among patients seeking aesthetic enhancement of their nose [5, 6]. Fillers are employed to straighten the nasal dorsum, correct asymmetries, and achieve tip elevation. Advances in techniques have further allowed fillers to support the nasal tip, elevate the lateral alar regions, and widen the internal nasal valve [7].

Another product for noninvasive rhinoplasty is botulinum toxin type A, which is used to reduce the appearance of nasal “bunny lines” caused by muscle activity, decrease nasal width during smiling by reducing muscle activity, and improve the appearance of a droopy nasal tip when injected into the depressor septi nasi muscle [8].

Among the nonsurgical methods, barbed lifting threads have emerged as valid and effective aesthetic tools in clinical practice [9]. Their use across various facial areas has advanced significantly, driven by improvements in both manufacturing techniques and clinical practices [10, 11, 12]. Manufacturers have developed long‐lasting absorbable threads that mitigate the complications associated with permanent threads while providing extended clinical benefits [13]. These threads have also expanded their use to nasal reshaping, offering a broader range of indications over time. Barbed threads not only provide lifting effects but also exhibit volumizing properties, enhancing their utility in nonsurgical rhinoplasty. They are utilized for nasal tip lifting, nasal dorsal narrowing, alar narrowing, and the correction of dorsal irregularities [14].

The aim of this study was to evaluate the clinical efficacy and safety of various nonsurgical rhinoplasty techniques, including the use of hyaluronic acid (HA) filler, botulinum toxin type A injections, and barbed lifting threads, in patients seeking nasal reshaping. The study compared the outcomes of these treatments, assessing their impact on nasal aesthetics and patient satisfaction, as well as adverse effects. Specifically, the study aimed to determine the most effective nonsurgical approach for achieving desired aesthetic results with minimal complications and downtime.

## 2 Materials and Methods

### 2.1 Patient Selection

A total of 85 patients were selected for this study between March 2022 and July 2022. Patients were considered eligible for the study if they had at least one of the following indications: prominent nasal hump, long nose with lack of tip support, hyperactive depressor septi, and lack of nasal tip projection. Subjects were not included if they had previously operated noses, previously treated noses with fillers or threads, or any autoimmune disease. Patients were offered treatment options of nasal thread lifting, nasal fillers, and botulinum toxin injections.

### 2.2 Treatment Groups

Patients were offered two treatment options:

1. Nasal threads followed by botulinum toxin type A (BTX) injections with or without HA filler 2 weeks later.
2. HA filler with BTX injections.

Patients were divided into three groups:

- Group 1 (“Filler + BTX”): HA filler and BTX injections (*n* = 63).
- Group 2 (“Threads + BTX”): Threads followed by BTX injections 2 weeks later (*n* = 9).
- Group 3 (“Threads + Filler + BTX”): Threads followed by BTX injections and HA filler 2 weeks later (*n* = 13).

### 2.3 Procedure Details

#### 2.3.1 Threads Treatment

The Sole Rhinoplasty kit by Aptos was used. It comprised five P (LA/CL) multidirectional barbed threads (USP 2/0, EP 3), each 120 mm in length. Additionally, it included five round tip hollow needles (20 G × 120 mm, straight), one round tip hollow needle (23 G × 80 mm, straight), one lancet point needle (18 G × 40 mm, straight), one lancet point needle (30 G × 13 mm, straight), and a removable needle attachment.

**FIGURE 1:**
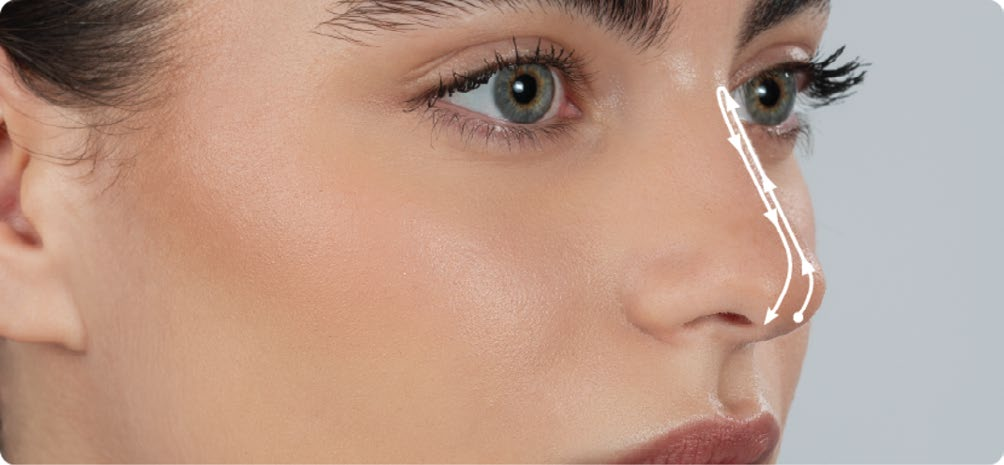
Rhinoplasty method using Aptos threads (The Sole Rhinoplasty kit).

Threads were inserted from the nasal tip through a puncture site made by an 18 G needle 2 mm below the nasal tip. Each thread was inserted and covered the length twice between the nasion superiorly and the nasal crest inferiorly (Figure 1).

#### 2.3.2 HA Filler

Restylane Lyft hyaluronic acid filler was utilized in all cases. In Group 1 (“Filler + BTX”), subjects received HA filler on the first treatment day and in Group 3 (“Threads + Filler + BTX”), 2 weeks after threads administration. In Group 1 (“Filler + BTX”), HA filler was applied to straighten the nasal dorsum and lift the tip through an intercolumellar injection and a tip bolus. The injection was performed using a 27 or 29 G needle, which was perpendicularly inserted over the nasion. Subsequently, the injection was carried out using a 25 G cannula introduced 2 mm below the nasal tip in the subcutaneous plane dorsally and between the medial edges of the lower lateral cartilages inferiorly. The volume of filler injected ranged from 0.5 to 1 mL.

In Group 3 (“Threads + Filler + BTX”), HA filler was administered 2 weeks after thread insertion. The dorsum was straightened using a 27 or 29 G needle, perpendicularly injected over the nasion. Additionally, the nasal tip was treated using a 25 G cannula to create a tip elevation by injecting up to 0.05 mL subdermally.

#### 2.3.3 Botulinum Toxin A Injection

For both groups 1 and 2, 4 IU of BTX (Allergan) were injected at the lower edge of the columella to reduce the activity of the depressor septi. In Group 1 (“Filler + BTX”), subjects received BTX on the first treatment day and in Group 3 (“Threads + Filler + BTX”), 2 weeks after threads administration.

### 2.4 Evaluation and Statistical Analysis

Patient satisfaction was evaluated using the FACE‐Q questionnaire for the nose before treatment, 1 month and 1 year after treatment (short‐term and long‐term assessment). The nose FACE‐Q module is a part of the FACE‐Q scales, it consists of 10 validated questions concerning patient's satisfaction with nose and four questions about adverse effects regarding the nose. For each question, a 4‐point scaling is used, with 1 being the minimal score for each question, and 4 being the maximal assessment. Individual scores were calculated as total out of 40 for patient satisfaction questionnaire or 16 for adverse effects. Nose adverse effects evaluation using FACE‐Q was performed by study subjects 48 h and 1 week after treatment for the purpose of safety assessment. Patients receiving threads treatment assessed the adverse effects 48 h and 1 week after the thread treatment but not after the additional treatment (BTX and, if needed, HA filler) 2 weeks later.

Statistical analysis was conducted using SPSS software. Nonparametric tests were used due to the non‐normal sample distribution. The Wilcoxon signed‐rank test was used for intragroup comparisons, and the Kruskal–Wallis test was employed for intergroup comparisons, with a *p* < 0.05 considered statistically significant.

## 3 Results

### 3.1 Study Subjects

A total of 85 patients were enrolled in the study: 17 men (20.0%) and 68 women (80.0%) 18–45 years old, mean (±SD) age was 29.3 ± 6.7 years. Descriptive statistics on patient demographics across treatment groups is shown in Table 1. Overall, patient age and gender distribution was similar in the three groups.

**TABLE 1:** Patient demographic characteristics.

Parameter/Group	Filler + BTX (*N* = 63)	Threads + BTX (*N* = 9)	Threads + Filler + BTX (*N* = 13)	Total (*N* = 85)
**Age, years**				
Mean ± SD	29.9 ± 6.8	27.2 ± 5.7	28.3 ± 7.0	29.3 ± 6.7
Median (IQR)	28 (9)	26 (11.5)	27 (11.5)	28 (7)
Min–Max	18–45	20–36	18–41	18–45
**Gender**				
Male, *n* (%)	13 (20.6)	2 (22.2)	2 (15.4)	17 (20.0)
Female, *n* (%)	50 (79.4)	7 (77.8)	11 (84.6)	68 (80.0)

*Note:* Filler + BTX, HA fillers and botulinum toxin type A injections; Threads + BTX, thread treatment, followed by botulinum toxin type A injections; Threads + Filler + BTX, thread treatment followed by botulinum toxin type A injections and HA fillers.

Abbreviations: IQR, interquartile range; Max, maximum; Min, minimum; *N*, number of subjects; SD, standard deviation.

### 3.2 Efficacy of Treatments

The efficacy of the treatments was evaluated using the FACE‐Q scores for nose 1 month and a year posttreatment. Nose FACE‐Q scores before treatment, 1 month, and a year posttreatment are summarized in Table 2. At baseline, all groups showed similar nose FACE‐Q scores with overall mean (±SD) of 17.8 ± 3.6, supposing similar level of concern regarding their nasal shape. No statistical difference between the three treatment groups was observed at baseline (Kruskal–Wallis test, *p* = 0.489).

**TABLE 2:** FACE‐Q nose scores for patient satisfaction 1 month and a year after treatment across treatment groups.

FACE‐Q score/Group	Filler + BTX (*N* = 63)	Threads + BTX (*N* = 9)	Threads + Filler + BTX (*N* = 13)	Total (*N* = 85)
**Baseline**				
Mean ± SD	17.8 ± 4.0	16.8 ± 1.8	18.0 ± 2.3	17.8 ± 3.6
Median (IQR)	19 (7)	18 (3)	18 (3)	19 (7)
Min–Max	11–24	14–19	15–21	11–24
**1 month posttreatment**				
Mean ± SD	33.4 ± 1.9	31.9 ± 2.5	36.2 ± 2.6	33.7 ± 2.4
Median (IQR)	33 (6)	34 (5)	36 (5)	33 (4)
Min–Max	32–37	29–34	32–39	29–39
*p*‐value (vs. baseline)^a^	< 0.001	0.007	0.001	
*p*‐value, group comparison	1.000^c^		0.001^b^	
			0.005^c^	
**1 year posttreatment**				
Mean ± SD	22.9 ± 3.0	25.1 ± 2.9	30.0 ± 0.7	24.3 ± 3.8
Median (IQR)	23 (4)	24 (4.5)	30 (1)	24 (5.5)
Min–Max	16–29	23–30	29–31	16–31
*p*‐value (vs. baseline)^a^	< 0.001	0.007	0.001	
*p*‐value (vs. 1 month)^a^	< 0.001	0.007	0.001	
*p*‐value, group comparison	0.32^c^		< 0.001^b^	
			0.021^c^	

*Note:* Filler + BTX, HA fillers and botulinum toxin type A injections; Threads + BTX, thread treatment, followed by botulinum toxin type A injections; Threads + Filler + BTX, thread treatment followed by botulinum toxin type A injections and HA fillers.

Abbreviations: IQR, interquartile range; Max, maximum; Min, minimum; *N*, number of subjects; SD, standard deviation.

^a^ FACE‐Q scores comparison, Wilcoxon signed‐rank test.

^b^ FACE‐Q score compared to the Filler + BTX group, Kruskal–Wallis test.

^c^ FACE‐Q score compared to the Threads + BTX group, Kruskal–Wallis test.

One month posttreatment, the FACE‐Q scores in all the three groups statistically significantly higher than at baseline (Table 2), in subjects treated with Filler + BTX and Threads + BTX, the mean nose FACE‐Q scores amounted to 33.4 ± 1.9 and 31.9 ± 2.5, respectively. Comparison of the FACE‐Q scores in three treatment groups revealed statistically significant difference between the groups (Kruskal–Wallis test, *p* = 0.001). In the Threads + Filler + BTX group, the mean (±SD) nose FACE‐Q score was 36.2 ± 2.6 which was statistically significantly higher compared to both Filler + BTX and Threads + BTX groups (Table 2).

The long‐term efficacy assessment showed a decrease in nose FACE‐Q score in the Filler + BTX group to 22.9 ± 3.0 one‐year posttreatment. Similarly, the Threads + BTX group showed a decrease to 25.1 ± 2.9 at 1 year. However, the Threads + Filler + BTX group exhibited a smaller reduction in efficacy, with scores decreasing to 30.0 ± 0.7 at 1 year. The FACE‐Q score decrease 1 year after treatment was statistically significant when compared to the corresponding values 1 month posttreatment in all the three groups. However, in all three groups, the FACE‐Q scores remained statistically significantly higher than at baseline (Table 2). These findings indicate that the combination of threads, HA filler, and botulinum toxin results in higher patient satisfaction and improved aesthetic outcomes in both the short and long term.

One year posttreatment, the statistical difference in nose FACE‐Q scores between the three groups was retained (Kruskal–Wallis test, *p* < 0.001). The Threads + Filler + BTX group showed statistically significantly better outcomes than the Filler + BTX group and Threads + BTX group. The mean (±SD) nose FACE‐Q score in Threads + Filler + BTX group was approximately by 30% and 20% higher than that in the Filler + BTX and Threads + BTX groups, respectively, obviously due to stronger score decay during the year in these two groups. No significant difference between the Filler + BTX and Threads + BTX groups was observed at either 1 month or 1‐year posttreatment, indicating similar efficacy in patient satisfaction for these two groups.

**FIGURE 2:**
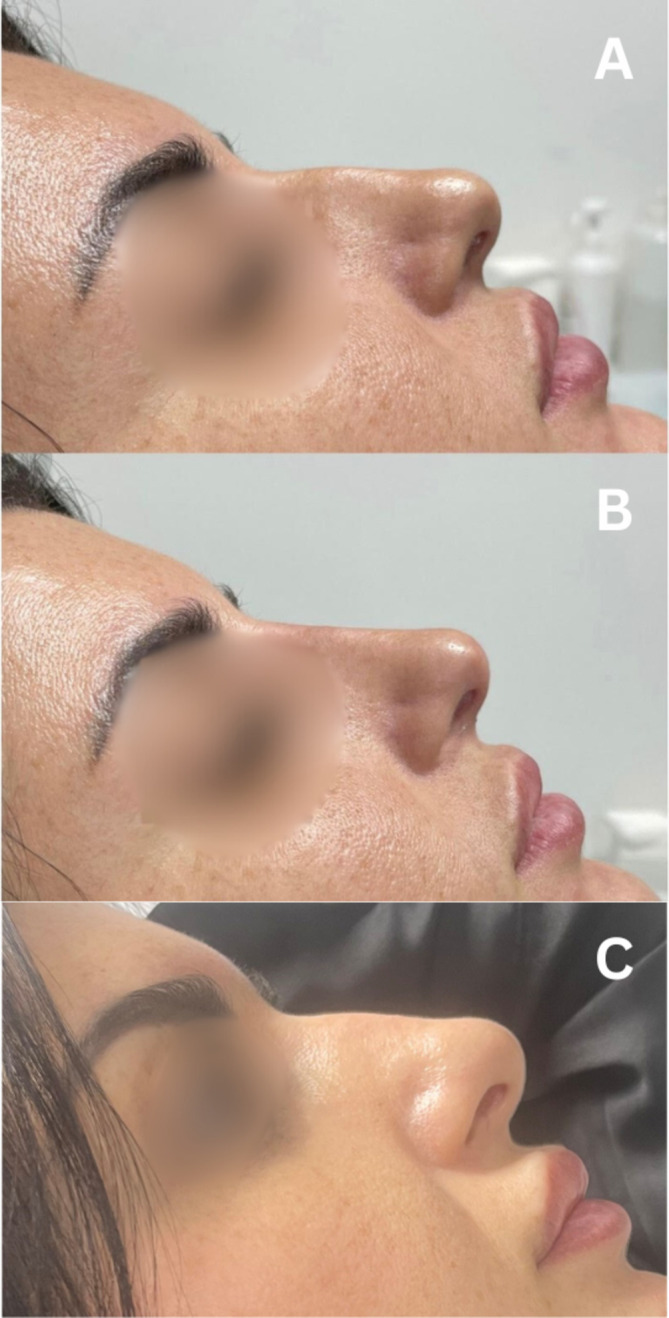
Patient from Group 2 (Threads + BTX) (A): (A) at baseline, (B) 1 month after treatment, and (C) after 1 year.

**FIGURE 3:**
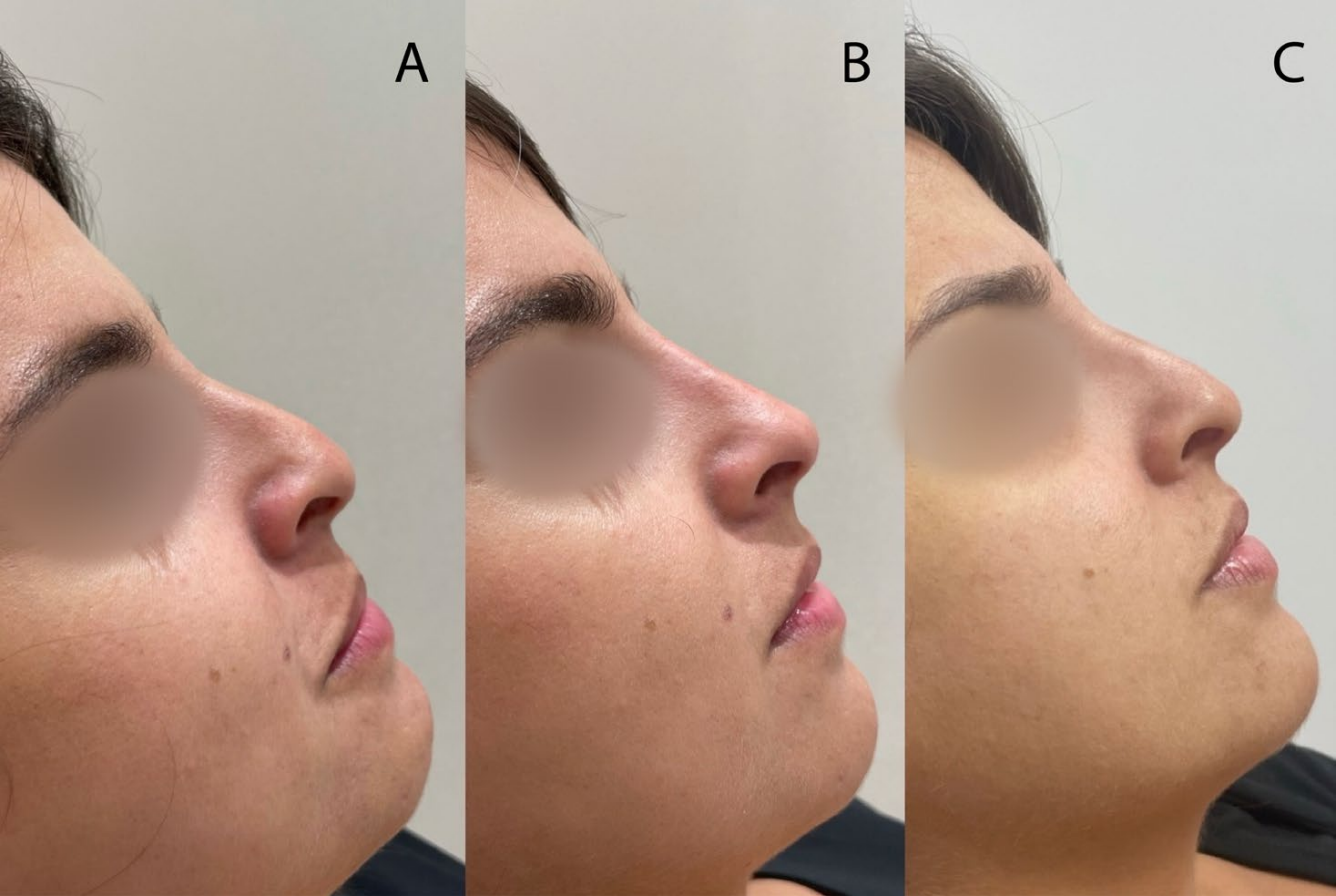
Patient from Group 3 (Thread + Botox + Filler): (A) at baseline, (B) 1 month after treatment, and (C) after 1 year.

**FIGURE 4:**
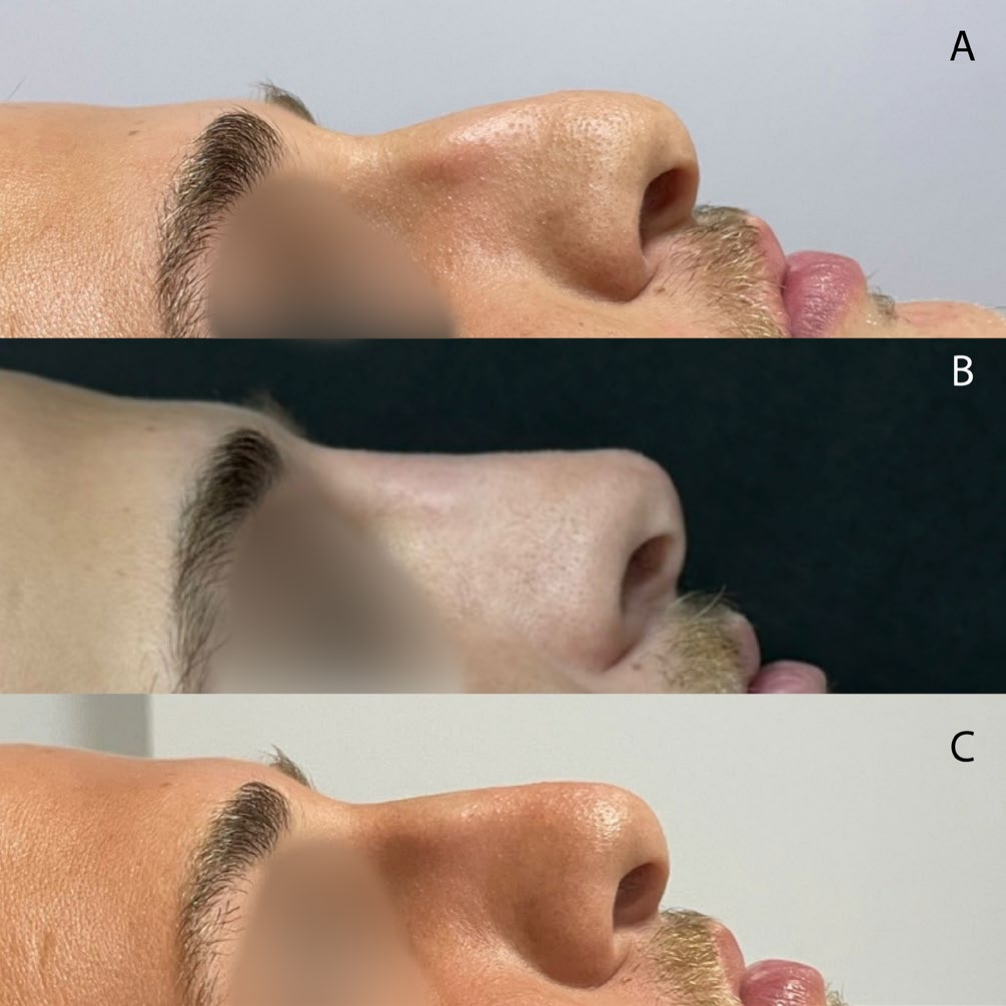
Patient from Group 3 (Threads followed by botulinum toxin type A injections and HA fillers 2 weeks later): (A) at baseline, (B) 1 month after treatment, and (C) after 1 year.

**FIGURE 5:**
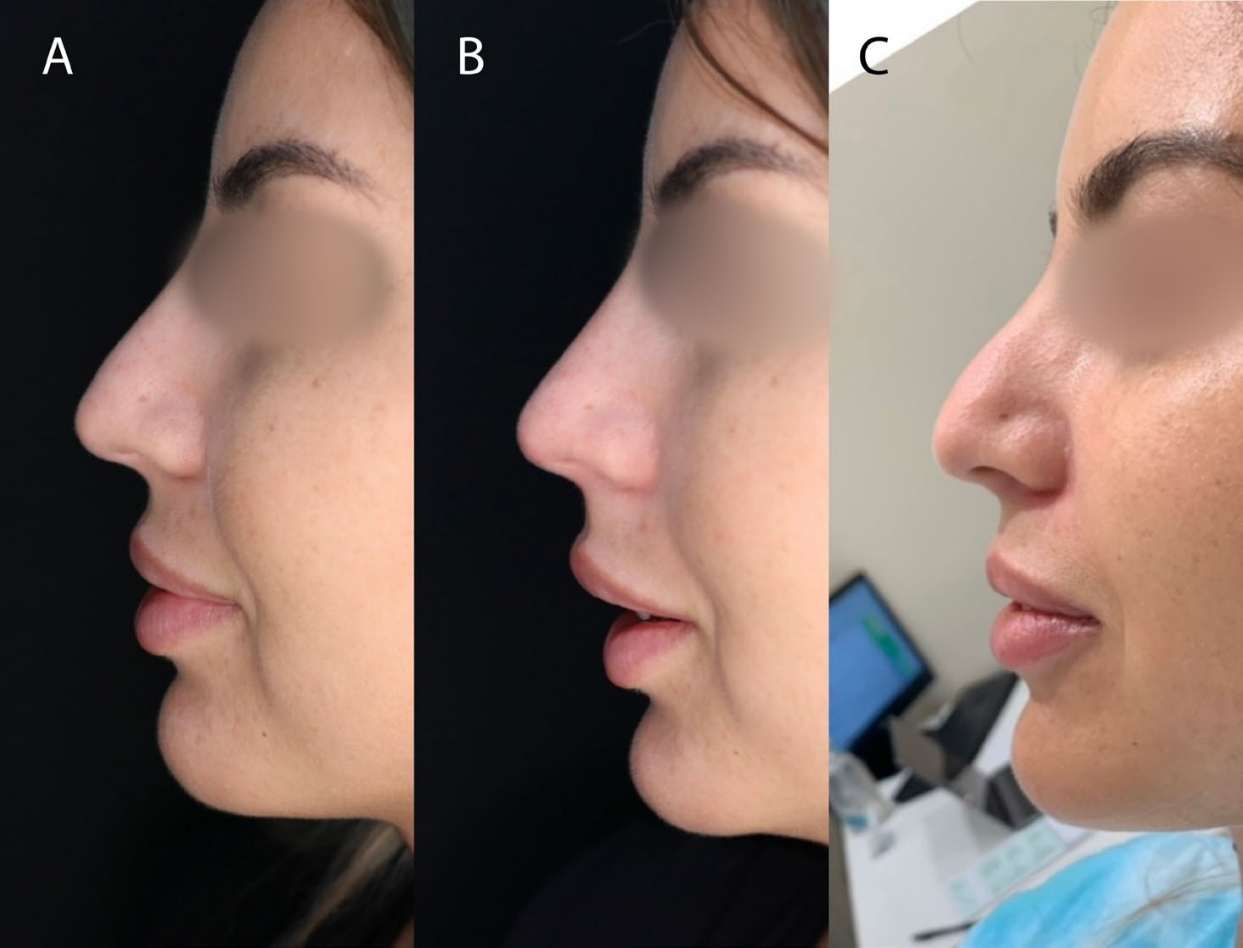
Patient Group 1 (HA fillers and botulinum toxin type A): (A) at baseline, (B) 1 month after treatment, and (C) after 1 year.

Thus, before treatment, all groups showed similar satisfaction level regarding their nasal shape. One month and 1 year posttreatment, patients in the Threads + Filler + BTX group expressed more satisfaction than those in the other two groups—the Filler + BTX and Threads + BTX groups, which exhibited similar levels of patient satisfaction at these time points (Figures 2, 3, 4, 5).

### 3.3 Adverse Effect Assessment With FACE‐Q

The nose adverse effects as per FACE‐Q questionnaire were assessed at two time points: 48 h and 1‐week posttreatment. Overall, at 48 h, the mean FACE‐Q adverse effect score (±SD) was 6.2 ± 1.6. Specifically, in the Filler + BTX group, the mean FACE‐Q adverse effect score was 5.7 ± 1.4, in the Threads + BTX group, it was 8.4 ± 1.4, and in the Threads + Filler + BTX group, it was 7.3 ± 1.0. At 48 h a statistically significant difference in FACE‐Q adverse effect score between the three treatment groups was observed (Kruskal–Wallis test, *p* < 0.001). As per group pairwise comparison, the adverse effects were statistically significantly more pronounced in the thread‐treated groups (Threads + BTX and Threads + Filler + BTX) compared to the Filler + BTX group due to the higher invasiveness of thread insertion (Table 3).

**TABLE 3:** FACE‐Q nose adverse effects 48 h and 1 week posttreatment across treatment groups.

FACE‐Q nose adverse effect score	Filler + BTX (*N* = 63)	Threads + BTX (*N* = 9)	Threads + Filler + BTX (*N* = 13)	Total (*N* = 85)
**48 h posttreatment**				
Mean ± SD	5.7 ± 1.4	8.4 ± 1.4	7.3 ± 1.0	6.2 ± 1.6
Median (IQR)	6 (3)	9 (3)	7 (1)	6 (2)
Min–Max	4–8	7–10	6–9	4–10
*p*‐value, group comparison	< 0.001^b^		0.003^a^	
			0.81^b^	
**1 week posttreatment**				
Mean ± SD	4.0 ± 0.4	4.0 ± 0.0	4.0 ± 0.0	4.0 ± 0.3
Median (IQR)	4 (0)	4 (0)	4 (0)	4 (0)
Min–Max	4–4	4–4	4–4	4–4
*p*‐value^c^	< 0.001	0.007	< 0.001	

*Note:* Filler + BTX, HA fillers and botulinum toxin type A injections; Threads + BTX, thread treatment, followed by botulinum toxin type A injections; Threads + Filler + BTX, thread treatment followed by botulinum toxin type A injections and HA fillers.

Abbreviations: IQR, interquartile range; Max, maximum; Min, minimum; *N*, number of subjects; SD, standard deviation.

^a^ Compared to the Filler + BTX group, Kruskal–Wallis test.

^b^ Compared to the Threads + BTX group, Kruskal–Wallis test.

^c^ FACE‐Q nose adverse effect score 1 week post‐treatment compared to 48 h posttreatment, Wilcoxon signed‐rank test.

One week posttreatment, the mean FACE‐Q adverse effect score in all the three groups reached similar level of 4 points, reflecting complete absence of adverse effects. By 1 week posttreatment, the FACE‐Q adverse effects scores statistically significantly reduced in all groups compared to those at 48 h (Table 3). No statistically significant difference between groups was observed (Kruskal–Wallis test, *p* = 0.840).

## 4 Discussion

Thread lifting has become an increasingly popular aesthetic procedure due to its effective lifting capabilities and reduced downtime compared to traditional surgical methods. Nonsurgical cosmetic interventions are highly sought after, as many patients have demanding schedules and prefer treatments that yield quick results with minimal complications and social downtime.

Our findings indicate that the combination of threads and botulinum toxin provides similar outcomes to fillers and botulinum toxin, with both groups showing statistically significant improvements at 1 month and 1 year posttreatment. While there were no significant differences in efficacy between these two groups, the superiority of barbed threads could be anticipated. The similarity in improvement can be attributed to the nature of the assessment tool, FACE‐Q, which provides a subjective evaluation of patient satisfaction but does not capture specific anatomical details such as nasal hump or tip. Consequently, future studies should incorporate objective assessment methods to capture these nuances more effectively.

Clinical observations suggest that comparable improvement observed in the threads + BTX group and Threads + HA filler group may be explained by the recurrence of the nasal hump 1 year after treatment. The high G prime properties of the HA filler likely contribute to superior dorsal support, maintaining nasal contour stability over time. In contrast, while patients in the thread‐treated group exhibited a clinically significant nasal tip lift at 1 year, often more pronounced than that in the HA + BTX group, the nasal hump recurred more frequently in this cohort compared to the HA + BTX group.

As noted, the addition of threads to HA filler and botulinum toxin yielded superior results, demonstrating statistically significant higher patient satisfaction in the Threads + Filler + BTX group both 1 month and 1 year after treatment. Specifically, 1 year after treatment a highly statistically significant difference between groups was observed in favor of the Threads + Filler + BTX treatment with a *p* < 0.001.

We acknowledge the statistical population imbalance observed in this study, which arose due to the natural distribution of patients during the data collection period. Clinically, this imbalance may be considered justifiable, as many patients demonstrate a preference for fillers and botulinum toxin injections owing to the shorter procedure time, reduced post‐procedural swelling, and the perception of these treatments as less invasive compared to threads. Additionally, threads are often regarded as a more advanced and potentially aggressive treatment option, which may influence patient decision‐making and account for the smaller group of threads‐treated individuals.

This study had limitations, in particular, the relatively small number of threads‐treated patients, and the lack of objective assessment using detailed measurements or 3D imaging software specifically designed for nasal shape evaluation. Future research should aim to incorporate reliable 3D programs for objective nasal shape analysis, capable of reporting skin surface changes and potential asymmetries, to enhance the precision and reliability of outcomes.

## 5 Conclusion

This study demonstrates that the combined modality of botulinum toxin, HA filler, and barbed threads is safe and provides superior short‐term and long‐term outcomes for nonsurgical rhinoplasty. The addition of HA filler and botulinum toxin at the nasion and nasal tip 2 weeks after thread insertion produced more rewarding results, enhancing both immediate and sustained aesthetic improvements. Although thread treatments initially had more pronounced adverse effects, these were temporary and resolved within a week. Despite the study's limitations, including small sample size in thread‐treated groups and the lack of objective assessment tools like 3D imaging, the findings support the effectiveness and safety of this combination approach. Future research should aim to incorporate advanced imaging techniques for more precise evaluations. Overall, the combined use of barbed threads, HA fillers, and botulinum toxin offers a promising and effective alternative to surgical rhinoplasty.

## Author Contributions

G. Ziade designed the study, wrote initial manuscript, and provided data interpretation. R. Saade contributed to data collection, interpretation, and manuscript writing. D. Daou performed statistical analysis. D. Karam assisted in data collection and study design. A. Bendito revised the manuscript, and M. Tsintsadze contributed to the study design.

## Ethics Statement

Study protocol was approved by the Ethics committee of Lebanese University. Informed consent, consent to reproduce their recognizable photographs, and consent for publication were obtained from every study subject.

## Conflicts of Interest

The authors declare no conflicts of interest.

## Data Availability

The data that support the findings of this study are available from the corresponding author upon reasonable request.
